# Circadian Rhythm Disturbances in Patients with Alzheimer's Disease: A Review

**DOI:** 10.4061/2010/716453

**Published:** 2010-09-02

**Authors:** Dawit A. Weldemichael, George T. Grossberg

**Affiliations:** Department of Neurology & Psychiatry, Saint Louis University School of Medicine, St. Louis, MO 63104, USA

## Abstract

Circadian Rhythm Disturbances (CRDs) affect as many as a quarter of Alzheimer's disease (AD) patients during some stage of their illness. Alterations in the suprachiasmatic nucleus and melatonin secretion are the major factors linked with the cause of CRDs. As a result, the normal physiology of sleep, the biological clock, and core body temperature are affected. This paper systematically discusses some of the causative factors, typical symptoms, and treatment options for CRDs in patients with AD. This paper also emphasizes the implementation of behavioral and environmental therapies before embarking on medications to treat CRDs. Pharmacotherapeutic options are summarized to provide symptomatic benefits for the patient and relieve stress on their families and professional care providers. As of today, there are few studies relative to CRDs in AD. Large randomized trials are warranted to evaluate the effects of treatments such as bright light therapy and engaging activities in the reduction of CRDs in AD patients.

## 1. Introduction

Circadian rhythms are the regular; near 24 hs rest/wake patterns seen in normal individuals. There is a progressive deterioration of circadian rhythms with aging. These include changes in the sleep wake cycle manifested by reductions in sleep quality and impairment in cognitive performance [1, 2]. Furthermore, exaggeration of age-related changes is seen in Alzheimer's Disease (AD) affecting as many as a quarter of patients during some stage of their illness. Few sleep questionnaires-based comparison studies have shown increased sleep disturbances in vascular cognitive disturbances compared to AD patients. CRDs also are noted to influence early recovery in poststroke patients [3].

Day-time agitation, night-time insomnia, and restlessness are among the common behavioral changes observed in AD. Nocturnal sleep disturbance is often accompanied by day-time napping, frequently in direct association with the extent of dementia [4].

Circadian Rhythm Disturbances (CRDs) in AD often present dramatically and are among the major triggers for institutionalization [5, 6]. They are also associated with shorter survival in long-term care residents [7, 8] and are a cause of physical and psychological burden for caregivers [9].

## 2. What does Cause CRDs?

The main reason for the alteration in sleep-wake cycle is related to alterations in the suprachiasmatic nucleus (SCN) and melatonin secretion [10]. Though not clear, genetic risk factors such as in AD patients who are negative for the APOE-4 allele have also been implicated in the development of sleep problems [11, 12].

The SCN of the anterior hypothalamus is the master clock controlling circadian rhythms in mammals. The SCN neurons exhibit near 24-hour electrical activity. The circadian influences of SCN neurons are distributed through different target organs by efferent neural and humoral signals particularly circulating melatonin. The nucleus controls melatonin secretion via a multisynaptic pathway producing changes in the biological clock, core body temperature, and sleep.

### 2.1. Biological Clock Changes

The activity of the SCN is regulated by environmental signals in a 24-hour cycle and runs autonomously. It resets on a daily basis via light inputs from the retina during the day and melatonin secretion during the night. These inputs are conveyed through the retinohypothalamic tract (RHT) resulting in oscillation of SCN and melatonin secretion [13–15]. The RHT originates from the glutamatergic ganglion cells of the retina. These cells express pituitary adenylate cyclase-activating peptide (PACAP) which elicits phase shift signals in the SCN. There is also a serotonergic input from the midbrain raphe nuclei providing an arousal-dependent influence that can reset the circadian clock and modulate the photic synchronization of the SCN [16, 17].

The SCN contains gamma-aminobutyric acid (GABA) and arginine vasopressin (AVP) neurons that send a direct inhibitory projection to the nucleus of the paraventicular nucleus of the hypothalamus [18–20]. This in turn activates melatonin secretion by the pineal gland. Circulating melatonin acts via MT1 and MT2 receptors and inhibits the firing of the SCN. Hence, it promotes sleep and resets the circadian pacemaker. Melatonin secretion rises 2 hours before regular bedtime and remains elevated throughout the night.

As mentioned above, alterations in the SCN and melatonin secretion are assumed to be the main reason for CRD in AD [10]. Further, there is loss of expression of AVP and MT1 receptors in the SCN. These in turn result in a reduced production of melatonin and disappearance of normal melatonin rhythm [21, 22]. The functional disruption of the SCN is even observed in the early stages of AD. The changes in the MT1 receptor explain why large studies have failed to show improvement in sleep-wake cycle after melatonin administration in AD patients [23].

### 2.2. Changes in Sleep Physiology

Certain sleep changes in AD seem to represent an exaggeration of changes that appear with normal aging. AD patients spend an increased amount of time in stage 1 sleep with increased number and duration of awakenings, compared to age-matched non-AD controls [24, 25].

With disease progression, it is also very difficult to separate EEG features of stage two sleep from stage 1 sleep. Sleep spindles and K complexes are poorly formed. They are also of lower amplitude, shorter duration, and much less numerous [26, 27]. The proportion of nonrapid eye movement (NREM) sleep increases with further disappearance of the true delta wave of slow wave sleep (SWS) [24–28].

The percentage of time spent in REM sleep, which remains stable with normal aging, is reduced in patients with AD [26]. A decrease in the mean REM sleep episode duration and REM sleep percentage can be due to degeneration of the nucleus basalis of Meynert. The nucleus normally exerts an inhibitory influence on the nucleus reticularis of the thalamus, the rhythm generator responsible for NREM sleep [29].

REM sleep also depends on the abundance and integrity of the cholinergic system. The cholinergic disturbance in AD is accompanied by worsening of REM sleep. In addition, many subcortical structures such as the basal forebrain, distal and superior raphe nucleus, and the reticular formation of the pons and medulla seem to be involved in the initiation of sleep and oscillation between REM and non-REM states. All of these structures may potentially be damaged by the degenerative changes which are part of AD. Their deterioration may explain many of the sleep architecture and rhythm changes in AD (see Table 1). 

### 2.3. Core Body Temperature Changes

In accordance with the endogenous circadian rhythm, melatonin also promotes sleep by lowering core body temperature via peripheral vascular dilatation [23]. Melatonin and core body temperature are inversely related. Lowest body temperature occurs 1-2 hours before awakening, generally between 4 and 5 AM. This corresponds to the maximum concentration of circulating melatonin levels. Core body temperature and melatonin are the two primary and measurable markers of the circadian cycle.

The core body temperature rhythm is generally accepted as yielding the best estimate of endogenous circadian amplitude and endogenous circadian phase in human subjects [30].

However, studies of circadian core body temperature rhythms in human subjects are complicated by the influence of many confounding variables (e.g., light, posture, meals, and social interaction) [31, 32]. Keeping these different variables constant while measuring core body temperature, studies examining differences and similarities in circadian disturbances in groups of normal elderly and patients with probable AD, as compared with a comparison group of young, normal volunteers have shown a reduction in endogenous circadian amplitude (ECA) and a delay in endogenous circadian phase (ECP) of core body temperature in patients with probable AD [33, 34].

Studies have demonstrated that normal aging is accompanied by a 40% decrease in endogenous circadian amplitude. Related studies showing a 50% reduction in amplitude in the AD group, as compared with normal young subjects could be interpreted as exaggerated or abnormal aging, rather than any discrete effect attributable to AD [30].

## 3. Common AD Symptoms Related to CRDs

Insomnia, nocturnal behavioral changes, and excessive daytime sleepiness, probably as a consequence of disrupted nocturnal sleep, are the common AD symptoms related to CRD. The actingout of REM sleep, often described as REM behavior disorder, is also briefly described here.

### 3.1. Insomnia

Insomnia in AD patients may have several etiologies. Though it may be primarily related to the underlying neurodegenerative changes producing CRD described above, other differential diagnostic issues need to be ruled out. For example, breathing problems during sleep, such as sleep apnea [35], with subsequent confusional arousal may be a possibility. A treatment trial with a continuous positive airway pressure (CPAP) breathing device has been reported to improve insomnia and even cognitive function in some patients.

Insomnia may also be secondary to restless legs syndrome (RLS), where patients feel a need to pace in the evening or night-time hours. Several studies have shown that the severity of leg discomfort follows a circadian rhythm, reaching a peak after midnight. Most patients report difficulty falling asleep or waking up shortly after sleep onset with unpleasant leg sensations. 

RLS, unlike the well-known mechanism of CRD, is mainly due to striatal dopamine depletion. Deficient iron transport and metabolism are also believed to result in RLS. If confirmed, serum ferritin levels may prove to be a useful laboratory marker [36].

Another common cause of insomnia especially in institutionalized or hospitalized AD patients may be secondary to noise in the environment and frequent room checks by staff. Bed checks are usually more frequent in incontinent patients to avoid decubitus ulcers.

Superimposed delirium can also interfere with restful night-time sleep. Simple measures such as treating urinary tract infections and pain, relieving fecal impaction, and reviewing the patient's current medications to determine if they may be promoting wakefulness or frequent awakenings [37] can result in drastic improvements of sleep and nocturnal agitation [38].

Macular degeneration and optic nerve degeneration are common conditions seen in AD which limit light input, further complicating circadian rhythms [39].

In patients with AD, other psychiatric comorbidities, and in particular depression and anxiety, are also important causes of insomnia. Treatment of these psychopathologies may potentially ameliorate insomnia in AD patients.

Adding to sleep difficulties can be the excessive intake of stimulants such as coffee, tea, or caffeine containing soft drinks. Theophylline, commonly used for broncho-pulmonary disease, is also a frequent cause of insomnia [40]. The cholinesterase inhibitors which are commonly prescribed in AD, and in particular donepezil, have been shown to occasionally cause insomnia [41, 42].

### 3.2. Hypersomnia

Excessive daytime sleepiness (Hypersomnia) is related to changes either in NREM (Idiopathic hypersomnia), REM sleep (narcolepsy without cataplexy) or could be central in origin (narcolepsy with cataplexy). Narcolepsy is seldom described as a degenerative disease of the hypocreatin-containing neurons in the lateral hypothalamus. It may be accompanied by cataplexy or sudden loss of muscle tone, vivid hallucinations, and brief periods of total paralysis and REM sleep at inappropriate times. Patients experience sudden urge to sleep which usually lasts for few seconds or minutes. A mutation in chromosome 6 (controls the Human Leukocyte Antigen) is seen in 90% to 100% of patients. Narcolepsy is usually seen in early adulthood and it is very uncommon in elderly patients with AD [43].

In elderly, hypersomnia is more frequently seen in patients with Parkinson disease (PD), Lewy body dementia (DLB), or Parkinson disease dementia (PDD) than in AD. A recently discovered hypothalamic wake-stimulating neuropeptide, hypocretin-1, has been hypothesized to be involved in consolidation of wakefulness. Lower hypocretin-1 levels may be permissive for increased wake fragmentation in AD resulting in daytime sleepiness. Stimulation of the hypocretin system may be a therapeutic means for improving daytime wakefulness in AD and in turn it helps sleep consolidation [44].

In AD patients, treatments of insomnia or medications used to control agitation at night are usually the most important causes of excessive daytime sleepiness (hypersomnia). In addition, the use of anticonvulsants and strongly anti cholinergic drugs such as antihistamines, antidiarrheals, and drugs used to reduce bladder irritability can cause daytime sedation and sleepiness [45].

Lack of engaging activities can also cause boredom and daytime sleepiness. At times, caregivers may promote daytime sleeping to ease their caregiving burden [46]. These in turn may result in night-time awakenings, further worsening the CRD [47–49].

### 3.3. Sundowning

Sundowning refers to agitation or a delirium-like state usually occurring late-afternoon/early evening or at night. Individual behavioral components of the condition which is frequently termed sundowning include loud vocalization, wandering, physical aggression, combativeness, maladaptive physical behaviors, and overall agitation [50–55]. These behaviors usually start at around 4 PM and last as late as 11 PM. However, some studies have reported the peak time of agitation as being in the afternoon, at approximately 2:30 PM, rather than in the early evening [56]. The prevalence of sundowning in AD ranges from 12% to 25 % [57].

The tendency for agitation in AD to occur at night suggests that some changes in sleep and wakefulness in the AD patient (chronobiologic changes) may reflect alterations in the body's ability to regulate the timing of certain physiological events. Common factors which cause late day confusion or “sundowning” include exhaustion, reduced lighting, and increased shadows with inability to separate dream from reality when sleeping. Agitation usually worsens when patients are transferred to a new environment or a new nursing home or when there is a change or shifting of caregivers. “Sundowning” typically peaks in the middle stage of the disease and then diminishes as AD progresses [58].

### 3.4. REM Sleep Behavior Disorder

RBD is classified as either idiopathic or secondary to some identifiable causes. In most cases, it is due to neurodegenerative diseases. Narcoleptic patients may sometimes experience RBD due to degeneration of hypocreatin-containing hypothalamic neurons. It may also be induced by some medications (e.g., sedative hypnotics, tricyclic antidepressants, anticholinergics, selective serotonin reuptake inhibitors). Two case reports of Rivastagmine-(cholinesterase inhibitor-) induced RBD have been shown in AD patients [59].

REM sleep behavior disorder involves violent physical activity during dreaming. It is due to failure of the mechanism that produces atonia during REM sleep. There is a high level of tonic and phasic muscle activity seen most typically in REM sleep. This is suggested by electromyographic (EMG) activity recorded from facial and limb muscles during sleep. This condition can be seen in AD patients' comorbid with synucleiopathies such as Parkinsons disease and/or Diffuse Lewy Body dementia. Patients with REM sleep behavior disorder may sustain injuries to themselves or their bed partner. The disorder usually occurs in reaction to vivid dreams.

REM sleep behavior disorder should be distinguished from nocturnal paroxysmal dystonia (NPD) which is characterized by abnormal night-time movement of the trunk and head with alternating flexing and extension of the limbs. NPD is often considered to represent nocturnal frontal lobe epilepsy typically accompanied by abnormal slow wave and spike activity with paroxysmal intrusions in NREM, rather than REM sleep [61]. It is usually treated with anticonvulsants such as carbamazepine [62–64], unlike REM behavior Disorder which is usually treated with clonazepam [65].

## 4. Treatment Approaches

There is empirical evidence to support the use of behavioral and environmental strategies in alleviating sleep problems and night-time behavioral disturbances in patients with AD. There are also certain pharmacological approaches to treat CRD-related symptoms. However, one needs to keep in mind that certain medications used for this purpose (e.g., benzodiazepines) can worsen cognitive deficits and obstructive sleep apnea syndrome (a condition commonly associated with AD). In addition, some medications may also induce daytime sleepiness, resulting in night-time wakefulness.

### 4.1. Behavioral/Environmental Approaches

There are simple and effective interventions which should initially be tried to treat CRDs in AD patients. These include a regular activity schedule (e.g., serving dinner early), bed time routines, decreasing noise and night-time exposure to room level light, and limiting caffeine and alcohol intake [60]. Outpatients and long-term care residents with AD may spend too much time in bed and not enough time engaging in physical activities. This contributes to circadian rhythm abnormalities. Hence, physical activity may influence circadian rhythms. Mental, physical, and social activities to avoid boredom and daytime napping may be useful. Carvalho-Bos et al. used two-week actigraphic estimates of sleep-wake rhythm and investigated its association with a range of functional domains in 787 demented elderly women. They found that nocturnal restlessness is related to impairment in daily function and social interaction [66].

Typically, long-term care residents with AD are exposed to only a few minutes of bright light each day, even less than patients living in the community. Because light exposure is the strongest known “Zeitgeber” (time cue) in humans, lack of daytime light exposure may contribute to circadian dysregulation [67, 68]. In one randomized controlled trial, 36 community-dwelling patients with AD were studied using a sleep survey and wrist actigraphy. Cases were randomized to daily exercise plus 60 minutes daily of bright light therapy (BLT) versus controls for 2 months. There was a significant reduction in the number of night-time awakenings, total time awake at night, and depression (*P* < .05) in the treatment group. Patients were also followed for the next 6 months and benefits were maintained [69, 70].

In institutionalized patients, BLT has been shown to reduce daytime nap duration by 30 minutes during the treatment period (*P* < .004) [70–72]. However, the effect in another study was not sustained after the cessation of light therapy [69, 73]. Another study has demonstrated modest benefits of bright light for two weeks on actigraphic measurements of activity [74–76]. A long-term (5 years) double blind, placebo-controlled randomized trial performed on 189 residents of 12 group care facilities in Netherlands has shown another modest benefit of bright light therapy in improving some of the cognitive and noncognitive symptoms of dementia. This effect is more attenuated when combined with melatonin administration [77, 78]. In summary, regular exposure to bright light may be effective for day/night reversal problems [73, 79–81]. If the patient has RBD, removing potentially dangerous items from the bedroom and placing a soft mattress next to the bed may also help to ensure safety.

Clinical experience-based data also suggest that some AD patients with obstructive sleep apnea syndrome, treated with CPAP, may benefit from improved sleep consolidation [82–84]. It is recommended that behavioral and environmental techniques should be tried first before resorting to pharmacologic approaches.

### 4.2. Pharmacological Approaches

There are various medications to treat insomnia, behavioral dyscontrol, and nocturnal agitation/wandering, excessive daytime sleepiness, REM sleep behavior disorder, and restless legs syndrome. Medications should always be combined with environmental/behavioral modifications. The choice of agent to use and dosing must be individualized. It is also the responsibility of the clinician to consider potential side effects and drug interactions.

Insomnia is a common problem which if left untreated/undertreated may exacerbate the cognitive and non cognitive symptoms of AD. Commonly used medications to promote sleep include trazodone, zolpidem, sedating antidepressants such as mirtazapine, less commonly, low doses of sedating atypical antipsychotics such as quetiapine, and chloral hydrate. (Refer to Table 2 for dosing). In considering the use of antipsychotics in the AD population, the FDA-issued warnings about increased risks of mortality, stroke, and stroke-like events need to be considered as part of risk/benefit calculations. As much as possible it is best to avoid benzodiazepines as they may worsen cognitive functions in AD patients. They can also impair balance and increase the risk of falls.

Nocturnal agitation/wandering, physical and verbal aggression, and hallucinations can also be treated pharmacologically after ruling out other medical and environmental causes and a trial of behavioral techniques. Atypical antipsychotics [85–87], anticonvulsants, short-acting benzodiazepines such as lorazepam or longer-acting agents (e.g., clonazepam), and trazodone may be effective measures. (Refer to Table 2 for dosing).

Cholinesterase inhibitors such as donepezil, rivastigmine, and galantamine may improve nocturnal behaviors over time [88, 89]. Cholinesterase inhibitors may significantly decrease evening-time hallucinations seen in AD patients [89]. Because of the dependence of REM sleep on the integrity of the cholinergic system, theoretically, one may predict increased REM sleep in cholinesterase inhibitor-treated AD patients [90]. Studies have found that rivastigmine increases the density of REM sleep in normal subjects [91]. However, cholinesterase inhibitors, especially donepezil, are also known to increase vivid dreams which subsequently may result in further disturbance in sleep [92, 93]. A double-blind placebo-controlled trial done by Grossberg et al. has shown that AD patients who received memantine performed better on behavioral outcomes compared to placebo [94].

Clonazepam, started at a low dose of 0.25 mg at night, is proven to be effective in the treatment of REM sleep behavior disorders [65]. Melatonin [95, 96] or pramipexole [97] can also be used in such cases although they are not as effective as clonazepam.

Although their use at higher doses is limited because of their potential for cardiovascular side effects in elderly, lower doses of methylphenidate or modafinil can be used if excessive daytime sleepiness is a problem. As usual, the risk/benefit ratio of these agents needs to be considered and discussed with patient/family before initiating these medications. It is also worth noting that stimulants may worsen some features of “sundowning”, for example, hallucinations.

Restless leg syndrome is a common condition which is not uncommonly seen in AD patients and which can further complicate CRD. Pramipexole 0.25 mg p.o. hs or ropinirole 0.5 mg p.o. hs are the commonly prescribed medications for this condition. The stimulating dopaminergic effect of these medicines can worsen sleep disturbance in some patients [65].

## 5. Recommendations/Conclusions

There are several causes of and contributors to CRD in AD. It is always important to rule out other potentially treatable comorbidities such as depression and delirium.

Caregiver understanding of the nature of AD and the implementation of environmental and behavioral techniques can reduce both patient and caregiver distress and may decrease hospital readmissions and delay institutionalization. We are beginning to understand the neuroanatomic, neurochemical, and endocrine underpinnings of CRDs in AD and their impact on sleep/wake disturbances as well as common behavioral abnormalities. At present, there is no definitive treatment to address the neurodegenerative changes underlying CRDs in AD. However, current therapies, whether behavioral/environmental or pharmacotherapeutic, can provide symptomatic benefits for patients and relieve stress on their family or professional caregivers.

Large controlled studies are needed to evaluate the effects of treatments such as BLT and engaging daytime activities in the reduction of CRDs and resultant sleep/wake cycle and behavioral disturbances in AD patients.

## Figures and Tables

**Table 1:** Changes in Sleep Parameters in AD.

Decrease in total sleep time
Decrease in efficiency of sleep with more fragmentation of sleep architecture
More time spent in stage 1 and stage 2 sleep and less time spent in deeper sleep
Increase in REM sleep latency and decreased REM sleep
Decreased density of eye movement activity

**Table 2:** Dosing schedule of medications used in CRD.

Symptom	Medication	Initial Dose	Titrating Schedule	Maximum Daily Dose
Insomnia	Trazodone	25 mg hs	25 mg increments q 3–5 days	50–100 mg
	Zolpidem	5 mg hs	5 mg increments q 3-4 days	5–10 mg
	Mirtazapine	15 mg hs	15 mg q week	15–30 mg
	Quetiapine	25 mg hs	25 mg increments q 3-5 days	25–100 mg
	Chloral hydrate	250 mg hs	250 mg increments q 5–7 days	250–1000 mg

Behavioral Dyscontrol	Memantine	5 mg am	5 mg increments q week	20 mg
	Donepezil	5 mg am	5 mg increments in 4 weeks	5–10 mg
	Rivastigmine Transdermal	4.6 mg od	9.5 mg 4 weeks later	9.5 mg
	Galantamine ER	8 mg od	8 mg increments q 4 weeks	16–24 mg
	Risperidone	0.25 mg hs	0.25 mg increments q week	0.5–1.5 mg
	Olanzapine	2.5 mg hs	2.5 mg increments q week	5–10 mg
	Carbamazepine (check level)	100 mg hs	100 mg increments q 3-5 days bid or tid	600 mg
	Oxcarbazepine	300 mg hs	300 mg increments q week	2400 mg
	Divalproex ER (check level)	125 mg hs	125 mg increments q 3-4 days	1500 mg

Excessive Daytime Sleepiness	Methylphenidate	2.5 mg am	2.5 mg increments am and early pm	20 mg
	Modafinil	100 mg am	100 mg increments q week	200 mg

REM Sleep Behavior Disorder	Clonazepam	0.25 mg hs	0.25 mg increment q week	1 mg
	Melatonin	3 mg hs	add 3 mg if needed	6 mg

Adapted from Current management of sleep disturbances in dementia: [60].
